# CircLONP2 Accelerates Esophageal Squamous Cell Carcinoma Progression *via* Direct MiR-27b-3p-ZEB1 Axis

**DOI:** 10.3389/fonc.2022.822839

**Published:** 2022-07-05

**Authors:** Cailin Zhu, Weiyun Bi, Hongtao Li, Wen Wang

**Affiliations:** ^1^ Department of Thoracic Surgery, The First Affiliated Hospital of Xi’an Jiaotong University, Xi’an, China; ^2^ Department of Clinical Skills Training Center, XiJing Hospital, The Fourth Military Medical University, Xi’an, China; ^3^ Department of General Surgery, General Hospital of Lanzhou PLA, Lanzhou, China; ^4^ Department of General Surgery, People’s Hospital of Tongchuan, Tongchuan, China

**Keywords:** CircLONP2, ESCC, miR-27b-3p, ZEB1, proliferation, migration

## Abstract

Circular RNAs (circRNAs) are important mediators in esophageal squamous cell carcinoma (ESCC) carcinogenesis. We aim to explore the functions and mechanisms of circLONP2 in ESCC progression. The circLONP2 level was evaluated in ESCC samples and cell lines. The role and mechanisms of circLONP2 in ESCC proliferation and migration were demonstrated *in vitro*. We found that circLONP2 was upregulated in human ESCC and predicts poor overall survival (OS) and disease-free survival (DFS). CircLONP2 promotes ESCC aggressiveness by directly interacting with miR-27b-3p, thus upregulating the expression levels of its target gene ZEB1 by suppressing miR-27b-3p activity. Therefore, we demonstrated that circLONP2/miR-27b-3p/ZEB1 axis promotes ESCC metastasis *via* regulating epithelial-to-mesenchymal transition (EMT)-related proteins. CircLONP2 may serve as an oncogenic circRNA and as a prognostic biomarker in ESCC progression.

## Introduction

Esophageal squamous cell carcinoma (ESCC), which is the major type of esophageal cancer among Chinese patients, ranks one of the most common malignancies in the digestive system (1, 2). Despite the advanced diagnostic technologies and promising therapies, the overall survival (OS) rate is still far from satisfactory, which brings a tremendous burden (3). Hence, it is urgently needed to identify new biomarkers for ESCC diagnostic and find out efficient therapeutic strategies for ESCC patients.

Circular RNAs (circRNAs), which consist of a circular configuration *via* a typical 5′ to 3′-phosphodiester bond, are recently recognized as a new member of non-coding RNA with more stable structures and specific functions (4, 5). It is well studied that circRNAs are dysregulated in many kinds of cancerous tissues, as well as cancer cell lines. CircRNAs participated in cancer cell proliferation, invasion, migration, and differentiation (4, 6, 7). The most fully explored molecular function of circRNAs in cancer progression is to act as a molecular sponge to sequester miRNA molecules, thus preventing the targeted mRNA from being degraded by microRNAs (miRNAs). For instance, stable overexpression of ciRS-7 has been identified in many forms of cancer tissues and cell lines, and the presence of ciRS-7 can upregulate the expression levels of miR-7 target genes by suppressing miR-7 activity (7, 8). CircACVR2A suppresses proliferation and metastasis of bladder cancer cells by sponge miR-626, thus regulating EYA4 expression (9). Recently, the expression profile of circRNAs has been studied in ESCC patients and demonstrated hsa_circ_0001946 and hsa_circ_0043603 as potential diagnostic biomarkers in plasma and secreted by exosomes in ESCC patients. Moreover, hsa_circ_0001946 could act as a prognostic biomarker in ESCC tissues (10). Other studies also indicated several important dysregulated circRNAs in ESCC, such as circGSK3β, which was associated with metastatic ability of ESCC by enhancing β-catenin signaling (11); hsa_circ_0006168, which was mediated mTOR signaling to handle the progression of ESCC (12); and ciRS-7, which was interacted with MAGE-A family, HOXB13 protein, and epidermal growth factor receptor (EGFR) signaling to manipulate the malignant phenotype of ESCC (13–15). Nevertheless, the role of circRNAs in ESCC is still largely undiscovered, which provides new opportunities and challenges for the future studies of ESCC. Previous studies reported that circLONP2 enhances colorectal cancer (CRC) invasion and metastasis (16). However, the role and function of circLONP2 in other types of cancers of the gastrointestinal system remain unknown.

In the present study, we explored and identified the carcinogenesis-related role of circLONP2 in ESCC. We first evaluated the expression of circLONP2 in human ESCC tissues and matched normal tissues and showed that patients with recurrence or with metastasis gained higher circLONP2 expression than those who are not suffering from recurrence or metastasis of ESCC. Moreover, upregulation of circLONP2 was associated with shorter OS and disease-free survival (DFS) of ESCC patients. Functional studies clarified that circLONP2 was mediated in ESCC cell proliferation and migration by sponge for miR-27b-3p and regulated its target gene ZEB1 expression. CircLONP2/miR-27b-3p/ZEB1 axis promotes ESCC progression *via* regulating epithelial-to-mesenchymal transition (EMT)-related proteins. Therefore, we believed that intervention of the circLONP2/miR-27b-3p-ZEB1 axis could be an effective method for ESCC treatment.

## Methods

### Cells and Treatments

Human normal esophageal squamous cell line (HEEC) and ESCC cell lines, including ECA109, EC9706, EC-1, ESC-410, and KYSE30, were cultured in Roswell Park Memorial Institute (RPMI) 1640 medium with 10% fetal bovine serum (FBS) (Gibco, NY, USA) and penicillin/streptomycin (Gibco, NY, USA). Silencing and overexpression of circLONP2 were constructed with LV-circLONP2 and sh-circLONP2 by lentivirus vectors (GENECHEM, Shanghai, China). Mimics and inhibitors of miR-27b-3p were synthesized (RiboBio, Guangzhou, China). The sequences were as follows: miR-27b-3p mimics Sense-UUCACAGUGGCUAAGUUCUGC, Antisense-AGAACUUAGCCACUGUGAAUU; mimics NC Sense-UUCUUCGAACGUGUCACGUTT, Antisense-ACGUGACACGUUCGGAGAATT; miR-27b-3p inhibitor GCAGAACUUAGCCACUGUGAA; Inhibitor NC-CAGUACUUUUGUGUAGUACAA.

### Patients and Samples

All primary ESCC tissue samples were obtained from patients during operations between January 2015 and December 2016 at the First Affiliated Hospital of Xi’an Jiaotong University (Xi’an, China). Patients who received antitumor treatment before surgery were excluded from this study. All patients provided clear pathological diagnoses, complete follow-up information, and informed consent. The ethical approval was supported by the Ethics Committee of the First Affiliated Hospital of Xi’an Jiaotong University.

### Proliferation Assays

3-(4,5)-Dimethylthiahiazo(-z-y1)-3,5-di-phenytetrazoliumromide (MTT), 5-bromo-2-deoxyuridine (BrdU), and colony formation assays were used to evaluate the proliferation property of circLONP2/miR-27b-3p-ZEB1 axis in ESCC. For MTT, after transfection for 72 h, cells (4,000 cells/well) were collected and cultured into 96-well plates using RPMI 1640 medium (100 μl/well). The MTT solution was added to corresponding wells at 0, 1, 2, 3, and 4 days and cultivated for 3 h. The optical density (OD) value was measured at 490 nm with a spectrophotometer (Elx800, BioTek, Winooski, VT, USA). For colony formation, cells were collected and cultured into six‐well plates (1,000–1,500 cells/well) for 14 days. Then, the cells were fixed and dyed, and the crystal violet-positive cells were counted.

### Migration Assay

The migration ability of the circLONP2/miR-27b-3p-ZEB1 axis in ESCC was tested by Transwell, using a 24-well insert with 8 μm of pore size. Cells (1 × 10^5^ cells/well) were collected and cultured into the top side with FBS-free RPMI 1640 medium, while on the lower side, 500 μl of RPMI 1640 medium with 30% FBS was added. Cells were fixed and stained 24 h later, and cells that penetrated to the underside of the membrane were counted. Wound-healing assays were performed to evaluate the cell migration ability of circLONP2. A cell monolayer was scratched to establish a cell-free region and observed cell migration into the wound after 24 h.

### RNA Extraction and Real-Time Quantitative PCR

Total RNA was isolated from human ESCC tissues and cell lines using TRIzol reagent (Invitrogen, Carlsbad, CA, USA) according to the manufacturer’s instructions. Reverse transcription of mRNA and miRNA was performed using random primers and stem-loop primers, respectively. RT-qPCR results were normalized to β-actin or U6. The primers used in this article are listed as follows: circLONP2, F-5′-GACTGAGAGAGAAGGCGCAC-3′, R-5′-TGGGTTGTTCACTCCCACAG-3′; β-actin, F-5′-GGGAAATCGTGCGTGACATTAAG-3′, R-5′-TGTGTTGGCGTACAGGTCTTTG-3′; miR-27b-3p, F-5′-CGGCAGTTCACAGTGGCTAA-3′, R-5′-CAGAGCAGGGTCCGAGGTA-3′; U6, F-5′-ATTGGAACGATACAGAGAAGATT-3′, R-5′-GGAACGCTTCACGAATTTG-3′; ZEB1, F-5′-AAAGATGATGAATGCGAGTC-3′, R-5′-TCCATTTTCATCATGACCAC-3′.

### Western Blotting

Total protein was extracted from ESCC cells by using radioimmunoprecipitation (RIPA) buffer (Beyotime Biotechnology, Jiangsu, China), containing a 1% protease inhibitor (Millipore, MA, USA). Each sample was adjusted to the same concentration by a bicinchoninic acid (BCA) assay kit (Beyotime Biotechnology, Jiangsu, China). The proteins with different molecular weights were separated by sodium dodecyl sulfate–polyacrylamide gel electrophoresis (SDS-PAGE), transferred onto polyvinylidene fluoride (PVDF) membrane (Millipore, MA, USA), and incubated with primary antibodies. Then the PVDF membrane was washed with PBS to remove the unbounded antibodies and then incubated with secondary antibodies (anti-mouse/rabbit IgG, horseradish peroxidase (HRP)-linked antibody, 1:1,000, Cell Signaling Technology, MA, USA), and finally visualized by enhanced chemiluminescence (ECL) chemiluminescent reagent (Millipore, MA, USA). The primary antibodies were listed as follows: ZEB1 (#3396, 1:1,000, Cell Signaling Technology, MA, USA), E-cadherin (#14472, 1:1,000, Cell Signaling Technology, MA, USA), Vimentin (#5741, 1:1,000, Cell Signaling Technology, MA, USA), and β-actin (#3700, 1:2,000, Cell Signaling Technology, MA, USA).

### RNA Pull-Down

For the assay of pulling down miR-27b-3p by circLONP2, whole-cell lysate from ESCC cells with circLONP2 stably overexpressed was mixed with a biotin-labeled circLONP2-specific probe and negative control probe at 4°C overnight. As for the determination of pulling down circLONP2 by miR-27b-3p, EC-9706-circLONP2 cells were transfected with either biotinylated wild-type (WT) or mutant miR-27b-3p mimics. Then the cells were collected and incubated with C-1 magnetic beads on the rotator at 4°C overnight. Then the prewashed streptavidin magnetic beads (Invitrogen, CA, USA) were added and incubated for 4 h at 4°C. The RNA reserved in the beads was extracted by TRIzol (Invitrogen, CA, USA) and further detected by RT-qPCR, using β-actin as an internal control.

### RNA Immunoprecipitation Assay

The RNA immunoprecipitation (RIP) assay was conducted using the Magna RIP RNA-bing Protein Immunoprecipitation kit (Millipore, USA) according to the provider’s protocol. Briefly, ESCC cell lysates were cultured with Dynabeads-coated IgG antibody (Millipore, USA) or AGO2 antibody (Cell Signaling Technology, USA) for 12 h at 4°C. The purified RNA was subjected to qRT-PCR to detect the enriched circLONP2 and miRNA.

### Luciferase Reporter Assay

The circLONP2 or ZEB1 3′ UTR sequences containing WT or mutant miR-27b-3p binding sites were synthesized and inserted into pmirGLO luciferase reporters and then co-transfected with miR-27b-3p mimics or control mimics into ESCC cells using Lipofectamine 2000. The luciferase activities were tested by the dual-luciferase reporter assay kit.

### Statistical Analysis

Student’s t-test was used for continuous data, whenever appropriate. ANOVA test was used for multiple comparisons with more than two groups, whenever necessary. Data are mean ± SD, and all experiments were repeated at least three times. A *p*-value of less than 0.05 is defined as statistical significance.

## Results

### CircLONP2 Was Upregulated in Human Esophageal Squamous Cell Carcinoma and Predicts Poor Overall Survival and Disease-Free Survival

**Figure 1:**
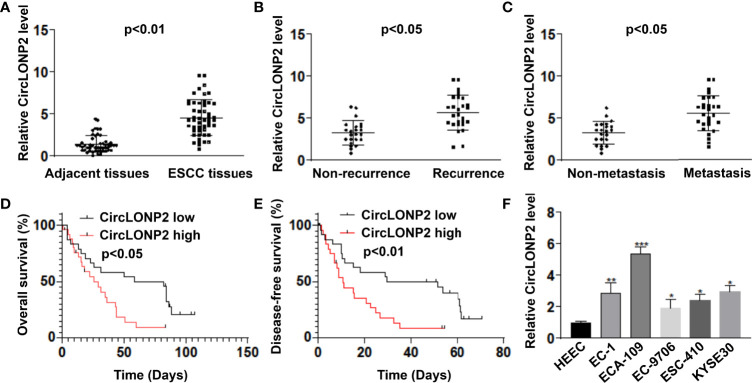
CircLONP2 was dysregulated in human esophageal squamous cell carcinoma (ESCC), and a higher level of circLONP2 predicts lower overall survival (OS) and disease-free survival (DFS). **(A)** CircLONP2 levels were elevated in ESCC tissues compared with paired non-tumor tissues as determined by RT-qPCR. Data are mean ± SD, n = 50. **(B)** Elevated mRNA levels of circLONP2 in recurrence patients than in patients without recurrence. **(C)** Elevated mRNA levels of circLONP2 in metastasis patients than in patients without metastasis. **(D, E)** Kaplan–Meier curve of circLONP2 depicting the OS and DFS of ESCC patients. **(F)** The expression circLONP2 was determined by RT-qPCR in cell lines. Data are mean ± SD. *p < 0.05, **p < 0.01, ***p < 0.001.

To figure out whether circLONP2 is dysregulated in ESCC, we first tested the expression of circLONP2 in ESCC tissues and paired non-tumor tissues by RT-qPCR. As shown in **Figure 1A**, the circLONP2 level was elevated in most ESCC tissues compared with their paired non-tumor tissues (n = 50, 1.40 ± 1.00 *vs.* 4.54 ± 2.14, *p* < 0.01), showing that upregulation of circLONP2 is a common phenomenon in ESCC patients. Then, we analyzed the clinicopathological data of each patient and found that patients with recurrence or with metastasis showed higher circLONP2 expression than those who is not suffering from recurrence (**Figure 1B**, 3.25 ± 1.41 *vs.* 5.65 ± 2.06, *p* < 0.05) or metastasis (**Figure 1C**, 3.25 ± 1.35 *vs.* 5.56 ± 2.12, *p* < 0.05). Moreover, upregulation of circLONP2 was associated with shorter OS and DFS (**Figures 1D, E**). All these data drive us to figure out the potential mechanism of circLONP2 in ESCC development. So we utilized cell line analysis to achieve this goal. As shown in **Figure 1D**, the RT-qPCR test for ESCC cell lines and the control cell line HEEC confirmed the higher expression of circLONP2 in ESCC cell lines (**Figure 1F**), suggesting that upregulated expression of circLONP2 may manipulate the progression of ESCC.

### CircLONP2 Is Essential for Esophageal Squamous Cell Carcinoma Cell Proliferation and Migration

**Figure 2:**
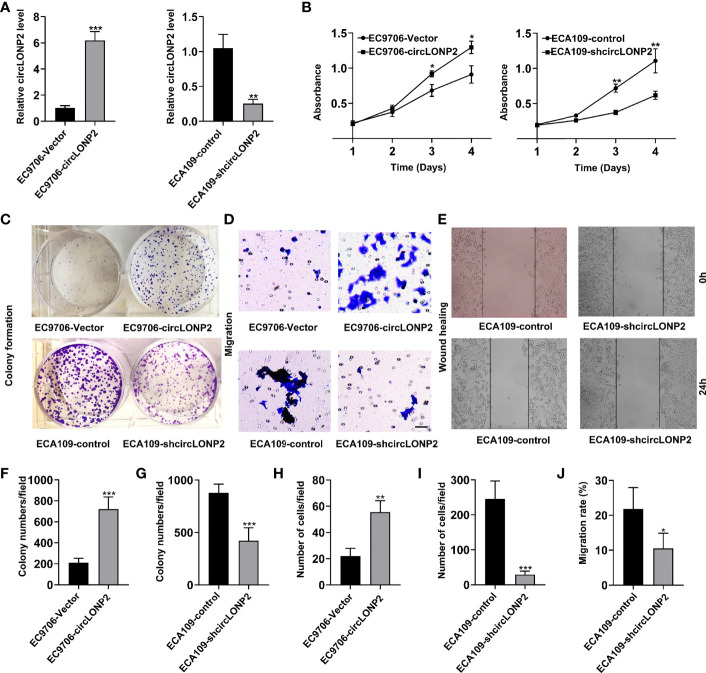
CircLONP2 is essential for esophageal squamous cell carcinoma (ESCC) cell proliferation and migration. **(A)** Construction of the circLONP2 overexpression cell line termed EC9706-circLONP2 and circLONP2 knockdown cell line, termed ECA109-shcircLONP2, as determined by RT-qPCR. Data are mean ± SD, **p < 0.01, ***p < 0.001. **(B)** EC9706-circLONP2 cells gained faster proliferation rate compared with EC9706-vector cells, whereas ECA109-shcircLONP2 cells showed lower proliferation rate compared with ECA109-control cells as indicated by BrdU assay. Data are mean ± SD, *p < 0.05, **p < 0.01. **(C, F, G)** Colony formation assay showed that EC9706-circLONP2 cells gained more colony numbers and larger colony clusters than EC9706-vector cells. In contrast, fewer colony numbers and smaller colony clusters were observed in ECA109-shcircLONP2 cells when compared with ECA109-control cells. Data are mean ± SD, ***p < 0.001. **(D, H, I)** Increased migration cells were observed in EC9706-circLONP2 cells when compared with EC9706-vector cells, while silenced circLONP2 could effectively inhibit the migration ability when compared with ECA109-control cells as determined by the Transwell experiment. Data are mean ± SD, ***p < 0.001. **(E, J)** Wound-healing assay of EC9706-circLONP2 cells and EC9706-vector cells. Data are mean ± SD, *p < 0.05.

**Figure 3:**
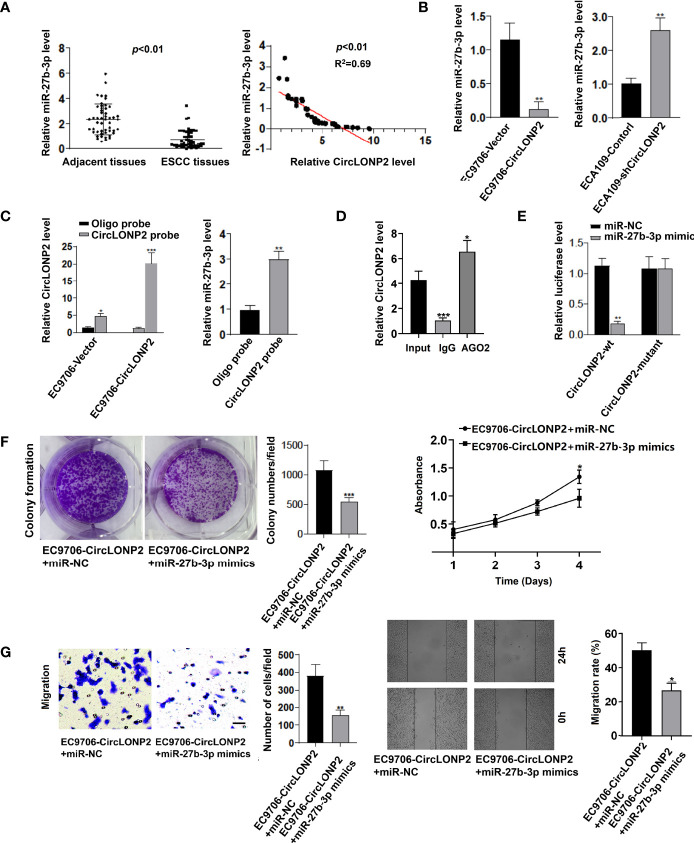
CircLONP2 promotes esophageal squamous cell carcinoma (ESCC) aggressiveness through directly interacting with miR-27b-3p. **(A)** MiR-27b-3p was universally decreased in ESCC tissues compared with paired non-tumor tissues as determined by RT-qPCR. Data are mean ± SD, n = 50; miR-27b-3p downregulation was negatively associated with circLONP2 expression. **(B)** Overexpression of circLONP2 in ESCC cells leads to a decrease in miR-27b-3p levels, while downregulation of circLONP2 in ESCC cells leads to an increase in miR-27b-3p expression as determined by RT-qPCR. Data are mean ± SD, **p < 0.01. **(C, D)** RNA pull-down and RNA immunoprecipitation (RIP) assays. Data are mean ± SD, *p < 0.05, **p < 0.01, ***p < 0.001. **(E)** Luciferase activity of Luc-circLONP2-WT or Luc-circLONP2-mutant in ECA109 cells co-transfected with miR-27b-3p mimics. Data are mean ± SD. **p < 0.01. **(F, G)** Colony formation assay and Transwell experiment of EC-9706 cells transfected with miR-27b-3p mimics. Data are mean ± SD. **p < 0.01, ***p < 0.001.

**Figure 4:**
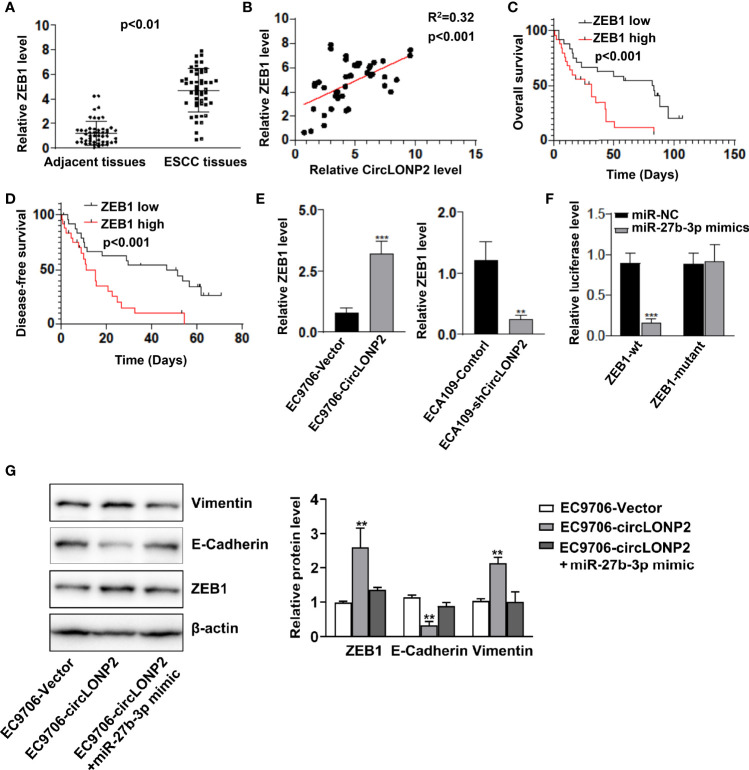
MiR-27b-3p regulates esophageal squamous cell carcinoma (ESCC) progression by directly targeting ZEB1. **(A)** mRNA level of ZEB1 in ESCC tissues compared with paired non-tumor tissues as determined by RT-qPCR. Data are mean ± SD, n = 50. **(B)** ZEB1 upregulation was associated with circLONP2 expression. **(C, D)** Kaplan–Meier curve of ZEB1 depicting the overall survival (OS) and disease-free survival (DFS) of ESCC patients. **(E)** Overexpression of circLONP2 in ESCC cells leads to an increase in ZEB1 levels, while downregulation of circLONP2 in ESCC cells leads to a decrease in ZEB1 expression. Data are mean ± SD. **p < 0.01, ***p < 0.001. **(F)** Luciferase activity of Luc-ZEB1-WT or Luc-ZEB1-mutant in ESCC cells co-transfected with miR-27b-3p mimics. Data are mean ± SD. ***p < 0.001. **(G)** Western blotting assay of epithelial-to-mesenchymal transition (EMT) markers in ESCC cells. Data are mean ± SD. **p < 0.01.

We then investigated the correlation between the circLONP2 expression and cell proliferation and migration of ESCC cells. Firstly, we utilized the EC9706 cell line, which exhibits the lowest circLONP2 expression among all the tested ESCC cell lines, to construct the circLONP2 overexpression cells, termed EC9706-circLONP2 (**Figure 2A**). We also used the ECA109 cell line, which exhibits the highest circLONP2 levels among ESCC cell lines, to construct the circLONP2 knockdown cell line, termed ECA109-shcircLONP2 (**Figure 2A**). We found that EC9706-circLONP2 cells gained a faster proliferation rate compared with EC9706-vector cells, as indicated by the MTT assay (**Figure 2B**), whereas ECA109-shcircLONP2 cells showed a lower proliferation rate compared with ECA109-control cells (**Figure 2B**). Accordingly, the colony formation assay showed that EC9706-circLONP2 cells gained more colony numbers and larger colony clusters than EC9706-vector cells (**Figures 2C, F**). In contrast, fewer colony numbers and smaller colony clusters were observed in ECA109-shcircLONP2 cells when compared with ECA109-control cells (**Figures 2C, G**). These results indicated that circLONP2 is essential for ESCC cell proliferation. Next, we evaluated the role of circLONP2 in ESCC cell migration by Transwell and wound-healing experiment. As expected, an increased number of migration cells were observed when circLONP2 is overexpressed, while silenced circLONP2 expression could effectively inhibit the migration ability of ESCC cells (**Figures 2D, E, H–8J**). These results showed that circLONP2 is also essential for ESCC cell migration.

### CircLONP2 Promotes Esophageal Squamous Cell Carcinoma Aggressiveness Through Direct Interaction With MiR-27b-3p

It is widely confirmed that miRNAs are key regulators involved in ESCC aggressiveness, and previous studies have demonstrated that circRNAs can serve as miRNA sponges to abrogate the functions of miRNAs (17–19). Therefore, we hypothesize that one possible mechanism of circLONP2 promoted ESCC progression may be owing to sponging miRNAs. Previous studies showed several potential target miRNAs of circLONP2, among which miR-27b-3p seems to be important in ESCC progression since it is also the possible upstream miRNAs of key oncogenic molecular ZEB1 (16, 20). To verify our hypothesis, we detected the miR-27b-3p expression in ESCC samples, EC9706-circLONP2 cells, and ECA109-shcircLONP2 cells. As seen in **Figure 3A**, miR-27b-3p was universally decreased in ESCC tissues, and its downregulation was negatively associated with circLONP2 expression. Similarly, overexpression of circLONP2 in ESCC cells led to downregulation of miR-27b-3p levels, while knockdown of circLONP2 in ESCC cells led to increased expression of miR-27b-3p (**Figure 3B**). All these results indicated that circLONP2 may interact with miR-27b-3p to regulate ESCC progression. To see whether circLONP2 can directly control miR-27b-3p expression, we performed RNA pull-down and RIP assays and showed that miR-27b-3p interacted with circLONP2 (**Figures 3C, D**). Moreover, using luciferase reporter assay, we also confirmed that miR-27b-3p mimics could successfully affect the relative luciferase activity when circLONP2-3′-UTR sequences contain WT miR-27b-3p binding sites but did not change so much the luciferase activity of circLONP2-3′-UTR sequences containing mutant miR-27b-3p binding sites (**Figure 3E**). Rescued experiments were used to evaluate whether miR-27b-3p could reverse the ability of circLONP2 to promote ESCC progression by upregulation of miR-27b-3p in the EC9706-circLONP2 cells. The data indicated that miR-27b-3p mimics could dramatically reverse the proliferation and migration in EC9706-circLONP2 cells (**Figures 3F, G**). Together, we showed that one possible mechanism of circLONP2 promoted ESCC progression could be owing to sponging miR-27b-3p.

### MiR-27b-3p Regulates Esophageal Squamous Cell Carcinoma Progression by Directly Targeting ZEB1

It is reported that ZEB1 is an essential factor with prognostic value in ESCC progression (21–23), and miR-27b-3p is a direct target of ZEB1 in diabetic nephropathy (20). We analyzed the expression of ZEB1 in ESCC samples and showed that ZEB1 mRNA is upregulated in ESCC tissues, and its upregulation was associated with circLONP2 high expression (**Figures 4A, B**). Moreover, we found that high expression of ZEB1 indicates unsatisfactory OS and DFS (**Figures 4C, D**). As expected, overexpression of circLONP2 in ESCC cells increased ZEB1 expression, while downregulation of circLONP2 led to ZEB1 downregulation (**Figure 4E**). Using luciferase reporter assay, we also confirmed that miR-27b-3p mimics could successfully affect the relative luciferase activity when ZEB1-3′ UTR sequences contain WT miR-27b-3p binding sites (**Figure 4F**). Because ZEB1 is a key regulator of EMT, we tested the EMT markers and found that upregulation of circLONP2 promoted EMT, as indicated by upregulation of Vimentin and downregulation of E-cadherin (**Figure 4G**). All these data indicated that circLONP2/miR-27b-3p/ZEB1 axis plays a key role in ESCC progression and could be a potential target for ESCC intervention.

## Discussion

ESCC accounts for approximately one-quarter million of the population every year and is the 5th leading cause of cancer-related death in China (24). The importance of circRNAs in human cancers had been paid much attention recently, and most researchers believed that modulation of circRNAs expression may serve as a novel diagnostic and therapeutic modality for cancers. In this study, we explored and identified that circLONP2 serves as an oncogenic circRNA and as a prognostic biomarker in ESCC progression. We clarified that circLONP2 was mediated in ESCC cell proliferation and migration by sponge for miR-27b-3p and regulated its target gene ZEB1 expression. Therefore, we believed that intervention of the circLONP2/miR-27b-3p-ZEB1 axis could be an effective method for ESCC treatment.

Previous studies reported that circLONP2 was significantly upregulated in human CRC, and circLONP2 enhances CRC invasion and metastasis through modulating the maturation and exosomal dissemination of miR-17 (16). However, the role and function of circLONP2 in other types of cancers remain unknown. These aroused our curiosity to explore the role of circLONP2 in other gastrointestinal cancers, such as ESCC. We first used 50 pairs of ESCC tissues and adjacent tissues to test whether circLONP2 is dysregulated in ESCC. The results showed that the circLONP2 level was elevated in most ESCC tissues, indicating that upregulation of circLONP2 is a common molecular event in ESCC patients. Kaplan–Meier (KM) curve revealed that a high circLONP2 level predicted unsatisfactory OS and DFS in ESCC progression. To seek molecular mechanisms of circLONP2 in ESCC progression, we construct the circLONP2 overexpression and downregulation cell lines and analyzed the proliferation and migration capacity of circLONP2 in ESCC by gain/loss-of-function studies. The results showed that circLONP2 could promote ESCC cell proliferation and migration. However, the mechanisms of this phenomenon are still unknown. Given that multiple miRNAs are involved in ESCC aggressiveness, circRNAs could serve as miRNA sponges to abrogate the functions of miRNAs in many cancers. So we hypothesize that one possible mechanism of circLONP2 promoted ESCC progression may be owing to sponging miRNAs. Previous studies showed that miR-27b-3p is a potential miRNA that could bind with circLONP2. MiR-27b-3p exerts tumor suppressor effects in ESCC by targeting Nrf2 (25). Moreover, miR-27b-3p is also a key regulator in chemotherapy-resistant ESCC cells (26). However, whether miR-27b-3p can be regulated by circLONP2 in ESCC remains unknown. Therefore, we first tested the level of miR-27b-3p in ESCC patients and analyzed the correlation between miR-27b-3p and circLONP2. The data showed that miR-27b-3p was widespread decreased in ESCC tissues, and its downregulation was negatively associated with circLONP2 expression. In addition, rescue experiments further indicated that miR-27b-3p mimics could dramatically reverse the proliferation and migration in EC9706-circLONP2 cells. All these data suggested that circLONP2 promoted ESCC progression could be owing to sponging miR-27b-3p. The next question is how miR-27b-3p regulates ESCC progression. We focused on its target gene ZEB1. ZEB1 is an essential factor with prognostic value in ESCC progression (21–23), as well as a direct target of miR-27b-3p (20). ZEB1 showed prognostic significance in ESCC tissues (21). A previous study confirmed that ZEB1-induced miR-99b/let-7e/miR-125a cluster promotes invasion and metastasis in ESCC (27). ZEB1 is also associated with Capn4-promoted ESCC metastasis (28). Recent research suggested repressing the biogenesis of circ-DOCK5 to facilitate metastasis in ESCC *via* a positive feedback loop with TGF-β (29). Higher expression of ZEB1 in ESCC patients was found in our study, and its upregulation was associated with circLONP2 expression. Luciferase reporter assay also confirmed that miR-27b-3p mimics could successfully affect the relative luciferase activity when ZEB1-3′-UTR sequences contain WT miR-27b-3p binding sites.

In a word, circLONP2/miR-27b-3p/ZEB1 axis plays a key role in ESCC progression and could be a potential target for ESCC intervention.

## Data Availability Statement

The original contributions presented in the study are included in the article/supplementary material. Further inquiries can be directed to the corresponding author.

## Ethics Statement

The ethical approval was supported by the Ethics Committee of the First Affiliated Hospital of Xi’an Jiaotong University. The patients/participants provided their written informed consent to participate in this study.

## Author Contributions

All authors significantly contributed to this study. WW designed the research, analyzed and interpreted the results, and prepared and approved the final version of the manuscript. CZ, WB, and HL performed the experiments and analyzed the data. All authors read and approved the final version of the manuscript.

## Conflict of Interest

The authors declare that the research was conducted in the absence of any commercial or financial relationships that could be construed as a potential conflict of interest.

## Publisher’s Note

All claims expressed in this article are solely those of the authors and do not necessarily represent those of their affiliated organizations, or those of the publisher, the editors and the reviewers. Any product that may be evaluated in this article, or claim that may be made by its manufacturer, is not guaranteed or endorsed by the publisher.

## Abbreviations

ESCC, esophageal squamous cell carcinoma; circRNAs, circular RNAs; OS, overall survival; DFS, disease-free survival; miRNAs, microRNAs; FBS, fetal bovine serum; OD, optical density; RIPA, radioimmunoprecipitation; SDS-PAGE, sodium dodecyl sulfate–polyacrylamide gel electrophoresis; WT, wild-type.
